# Collectanea Medica: Consisting of Anecdotes, Facts, Extracts, Illustrations, &c.

**Published:** 1822-11

**Authors:** 


### COLLECTANEA MEDICA:

CONSISTING OF

### ANECDOTES, FACTS, EXTRACTS, ILLUSTRATIONS, &c.

*Relating to the History or the Art of Medicine, and the Collateral Sciences.*

---

Floriferis, ut apes, in saltibus omnia libant,
Omnia nos, itidem, depascimur aurea dicta.

---

ART. I.—*On the Pathological Anatomy of the Human Brain and its Membranes.* By DAVID CRAIGIE, M.D. Lecturer on Anatomy and on Physiology, Edinburgh.

THE dura mater, the first and strongest membrane of the brain, may be considered as having a threefold use: 1st, that of an interior periosteum; 2d, that of a protecting membrane; 3d, that of influencing the circulation, by its sustaining the large venous trunks. It performs the part of a periosteal covering to the internal table of the cranium, to which it is connected by numerous arteries and veins, with a degree of firmness which is in general considerable, but is liable to great variation. These vessels, by which it is indirectly connected with the pericranium, seem in some instances to be capable of a morbid action, in consequence of injuries of the exterior envelope. This sympathetic action, as it has been named, has been fully considered, and very much over-rated, by Mr. Pott, who has laboured to impress the profession with the notion that the dura mater is always in an inflamed and subsequently sloughing condition, when the pericranium has been severely injured. There is, however, no direct proof that this is a uniform result. That it has existed in some few instances, we are perfectly willing to admit; but I am satisfied, from many observations on this point, that it takes place much less frequently than has been supposed. I have seen the pericranium detached for the space of two square inches, and a granulating process commence from the external table, but without the slightest proof of the dura mater being diseased. In the young subject it is by no means uncommon, when the pericranium is injured, to see the whole thickness of the cranium removed at the spot by the ulcerative absorption, and the wound begin to show the pulsating motion of the brain, and expel purulent matter at every motion of respiration: yet this morbid action will gradually disappear, and leave no trace of any great or important change in the dura mater having taken place. The effects, in short, which injuries of the pericranium produce on the vascular system of the dura mater, are very much varied by the age, disposition, and habits, of the individual; and, above all, by the treatment which is employed after the injury.

I have said that the dura mater, in the healthy state, adheres very firmly to the internal cranial surface; and it has been remarked, by Dr. Baillie and others, that the firmness with which it adheres may sometimes be so great as to amount to disease. I regret that observation forbids me to assent to this opinion; and I can see no reason why any degree of firmness with which it might adhere should be viewed as morbid. I am, on the contrary, inclined to think that the most frequent and ordinary effect of disease is to make it adhere with less firmness than usual; and I have, in the course of morbid investigations, met with at least one decided instance in which this appearance was remarkably distinct. A man, of about thirty-five years, who had received a slight blow on the head, began, some time after, to labour under pain of the whole syncipital region; and, in the course of a few months, had complete amaurosis of both eyes. Remedial treatment was employed under eminent surgeons without much benefit; and, in addition to his other maladies, he soon became epileptic. After suffering, in a hopeless manner, under his complaints for the space of six months, he fell into a comatose state, and died in a few days. Dr. Duncan, jun. and myself examined the body in the presence of some medical friends. On opening the head, we found that the internal table of the cranium did not adhere with the usual firmness to the dura mater, but instantly dropped off as soon as it was divided all round by the saw. The scull-cap, which was thus removed, was unusually thin through the greatest part of its extent; and this thinness was obviously produced by the removal of a part of the osseous structure of the internal table, by absorption perhaps. In some few points the original thickness of the cranium was left unchanged, so as to form the spicula of which pathographical writers have spoken in describing the appearances of the epileptic cranium. These spicula, however, were not produced, as has been generally supposed, by an augmentation of the ossific action, but, as I have already hinted, by the removal by absorption of the bone of the contiguous parts. No blood-drops appeared, either on the external surface of the dura mater or on the internal surface of the cranium; so that the usual arterial connexions of those parts were no longer present. The lateral and inferior regions of the cranium presented the same appearances; and the dura mater seemed to lie very loosely on all these parts of the basis of the cranium, to which it generally adheres with singular firmness. The other morbid changes were such as do not illustrate this state of the vascular connexions of the dura mater and cranium; but I may have occasion to allude to them when speaking of other pathological alterations in this region of the body.

The most usual morbid alteration to which the arachnoid membrane is liable, is the diminution of its transparency, without any change in its shining aspect. This is most remarkable in those parts of the membrane which correspond to the cerebral anfractuosities, where the arachnoid membrane has then a tinge of a bluish grey colour; but it is likewise sufficiently obvious on the apices of the convolutions, where it prevents the observer from perceiving the natural colour of these objects. The superior part of the hemispheres on each side of the median fossa is sometimes wholly occupied with this appearance; but, when it is present, it is always most conspicuous at the basis of the brain. In such circumstances, the arachnoid membrane is elevated and detached from the pia mater by the interposition of a watery fluid: its entire non-adherent surface has a gelatinous appearance; and some observers have thought that a semi-fluid substance resembling jelly was deposited beneath it; but, if a puncture or two be made, a considerable quantity of watery fluid is observed to escape; and the observation of this phenomenon is, in truth, a very good method of proving the existence of the arachnoid membrane.

In this instance, it may very justly be made the subject of inquiry, whether the fluid be derived from the external and adherent surface of the arachnoid membrane, or from the external surface of the pia mater. I am uncertain whether this has ever occupied the reflections of those who have had occasion to remark the appearance, and it would not, perhaps, be easy to devise expedients by which the question might be determined. If, however, we admit this fluid to derive its origin from the arachnoid membrane, the opinion must be held as a proof of the inconsistency of Bichat's reasoning concerning the nature of the arachnoid. Every effusion, whether inflammatory or serous, proceeding from a transparent membrane, takes place, we know, from the general characters of such membranes, at the internal or non-adherent surface of these organs. In this instance, however, the effusion takes place at an adherent or exterior surface of a membrane, which, he has laboured to show, possesses the characters of the transparent or serous membranes. We are, therefore, reduced to the alternative of either denying the arachnoid origin of the effusion, or of arranging this membrane in a different or individual class. If we still continue to consider the arachnoid membrane as analogous to the transparent envelopes in other regions of the body, we may then believe that the effusion is always induced by some peculiar action in the vascular system of the pia mater. Other reasons, however, influence me still more strongly in entertaining this opinion on the origin of the effusion beneath the arachnoid. Not only do we generally find the vessels of the pia mater more numerous and more distended in such circumstances, but we frequently, nay almost uniformly, observe an analogous change in a part which is a continuation and modification of the pia mater. I allude to the choroid plexus, which I have almost invariably, in such an event, found to exhibit in both ventricles a series of bodies resembling hydatids, about the size of a garden pea. Although these have been considered hydatids by many pathologists, yet I have very strong and satisfactory reasons for believing that they are the result merely of the natural structure of the vascular network being unfolded and distended by the effusion of a fluid, which is produced by the same action going on in a different part of the same membrane. At the posterior extremity of the mesolobe,* in the fissure between the posterior lobes of the brain proper and the cerebellum, the pia mater may be traced below the fornix into the middle or third ventricle, in the form of the *velum interpositum* or choroid web, and subsequently into the lateral ventricles in the form of the plexus choroides. When, therefore, from any cause, a morbid action

* This name, given by Chaussier, I prefer to the inapplicable circumlocution of *corpus callosum*.

is commenced, either on any part of the pia mater investing the convolutions or on that which is found in the central surface, this action may be propagated by continuity of surface from the one to the other, or *vice versa.* It is on this account, I conceive, that we frequently find the effusion between the arachnoid and the pia mater, accompanied by the distended state of the choroid cells, and even a quantity of fluid in the ventricles.

The latter circumstance, which very frequently attends the hydatoid appearance of the plexus, has led some practitioners to consider this as the acute hydrencephalus. It is hardly possible, however, to make a greater mistake in the pathology of those parts, than by confounding the two diseases. The effusion of which we have been speaking occurs in many subjects in whom there is but a very moderate affection of the head; and, at all events, very different symptoms from those which attend the acute hydrencephalus. When the system is under the influence of common contagious fever, it is exceedingly common to find this change in the head after death; and we generally observe that the last hours of existence have been spent in a state of stupor, or even coma. It is certain, however, that, in many cases in which no symptom of this kind could be recognized, the very same appearances were found on dissection. This diversity in the results of cases, apparently the same, has induced many pathologists to refer those examples of effusion which were indicated by no symptom during life, to the process of transudation after death. That this was an actual fact, was very probable for several reasons; but no direct proof was adduced to support the assertion. I consider myself somewhat fortunate in being able to furnish some very interesting observations on the point at issue, from very high and respectable authority. Joseph and Charles Wenzel, of Vienna, think that many examples of aqueous effusion in the ventricles do certainly take place after death; and, to confirm their opinion, detail the following cases:*

"On the 29th of May, 1801, four state criminals suffered at Mentz the punishment of decapitation by the guillotine. Thirty-five minutes after the punishment of the last, the body of the delinquent who was first beheaded was brought, along with the four heads, to the theatre of Ackermann, professor of anatomy. The cranium of three of these were immediately opened, and the brains submitted to careful examination, in the presence (it would appear) of the two Wenzels.

"On examining the brain of the individual who suffered last, a little pure fluid (lympha,) was found in the inferior and in the posterior corner of the left lateral ventricle. In the lateral ventricle of the right side, and in the fourth and fifth ventricles, not the slightest trace of fluid appeared. The cavity of the middle septum could not be detected. In neither of the cavities of the second brain could a drop of fluid be seen.

"The third brain presented the same phenomena as the one which had been first submitted to examination; but lymphy or watery fluid

* Josephus *et* Carolus Wenzel *De Penitiori Structura Cerebri Hominis et Brutorum.* Tubingæ apud Cottam. 1812. Fol. p. 199, *et seq.*

(lympha,) was observed at several spots between the arachnoid membrane and the pia mater. A small quantity of the same kind of fluid was found in the pericardium of the individual who was decapitated first.

"The brain of the fourth criminal was allowed to remain untouched till the morning of the following day, (May 30th,) when it also was subjected to examination, at an interval of about fifteen hours after death. The result was, that in the ventricle of the middle septum, in each division of the lateral ventricle on the left side, and in the fifth ventricle, the lymph was in general more copious than in the first and third brains."

From these observations, the Wenzels conclude that, in brains which are examined immediately after death, while the temperature of the body still continues, the presence and quantity of the fluid of the ventricles is in the same proportion as in brains which have been examined at a much later period. This fluid is, in truth, they remark, sometimes present, and sometimes it is not found; but, when it is, its presence seems to depend on no other cause than that which occasions the pericardial fluid, and what is found between the membranes of the brain; for the nature, condition, and end of both seem to be the same, and purely in consequence of the entire interruption of the circulation, and the extinction of the power of absorption after death, does that assume the appearance of an aqueous fluid, which, during life, was such a delicate vapour as to escape the eyesight.

These remarks, I conceive, go far to explain many of the cases of watery effusion into the ventricles; but they cannot, I am satisfied, account for the whole of these; for, if all the cases of the description we are considering depended on such a cause, we might expect to see fluid in every brain which we examined; and we should certainly observe a relation between its quantity and the time which has elapsed since the extinction of the vital actions. So far, however, as my observation goes, I am not aware that this has been established.

I believe, therefore, we must admit that effusion actually does take place between the membranes and on the central surface of the brain, without in every instance producing very conspicuous effects on other organs and functions. It is, in short, a condition of the cerebral membranes which may occur either with or without affection of the organs of sensation and voluntary motion; but I have, in general, remarked that, in those subjects in whom the comatose affection during life had not manifested itself, the effusion had taken place in a very slow and gradual manner. It is, in truth, in consequence of this slow and gradual change that the nervous system of the sensitive and locomotive apparatus is so little affected: it becomes, as it were, insensibly accustomed to the compression thus occasioned, and it is only when the effusion is extreme that any striking effect is experienced. Of this we have a remarkable instance in the phenomena of the chronic hydrencephalus, in which the same effusion takes place which we have now been considering. The serum is poured forth in great quantity between the arachnoid membrane and the pia mater, and in the central or interior surface of the brain, where the choroid plexus is likewise affected. Yet, although this state continues in some instances for many years, we rarely meet with any affection of the organs of sensation or locomotion. The last example of this disease, in which I had occasion to examine the cavity of the head, afforded a complete proof and illustration of the accuracy of these views. The subject was a child of about three years old, who had laboured under the symptoms of chronic hydrencephalus for about eighteen months or two years. The habit of this patient was strumous; he had the rachitic cranium; and the osseous texture was exceedingly soft, spongy, much lighter, and less compact then natural. Its size was considerably augmented, and the brain was proportionally distended. A very great quantity of fluid was found between the pia mater and the arachnoid membrane; the cells of the choroid were unfolded and distended; and the ventricles were considerably extruded by the fluid which they contained. Although it might have been presumed that this fluid would have speedily produced a fatal compression of the nervous system, yet the child existed in comparative health for many months, and died apparently in consequence of a disorganized state of the whole of the right lung and a considerable portion of the left. Every thing, in fact, that we yet know regarding this condition of the cerebral membranes, tends to establish this great general maxim,—that an effusion, nay, to speak more generally, any morbid change which takes place in a short time, and occasions a sudden compression of the brain, produces death very rapidly; whereas any change which comes on slowly, and produces its effects in a gradual manner and after a long period, seldom proves *suddenly* fatal. Under the former head we arrange the effusion which takes place in the course of contagious fever; that which is the result of acute hydrencephalus; that produced by the serous apoplexy, as I shall afterwards show; and, perhaps, an effusion which takes place in consequence of suppression of urine and traumatic gangrene. Of the latter we have an example in the effusion resulting from chronic hydrencephalus; in an effusion attending the development of morbid growths or tumors; and, perhaps, an effusion which frequently succeeds to the last stage of diseases of the heart.

As an effect of this effusion of fluid on the central surface of the brain, I must notice here a very interesting and curious change which sometimes takes place in the lucid septum. This consists in the progressive attenuation, perforation, and rupture of the septum, so as to form in it a considerable hole, by which the two lateral divisions of the central surface, called the ventricles, communicate directly and freely. At first the septum, thin as it is, becomes thinner, and actually translucent; very soon a number of minute perforations appear in it, of no determinate figure, but which, gradually enlarging, are separated from each other by thin capillary threads of the cerebral substance of which the septum is composed. In this state it looks like a small piece of network placed between the mesolobe and fornix; and a very trifling force applied to it will destroy the interlacement of cerebral threads, and produce a hole of an elliptical figure, the larger diameter of which will be antero-posterior. I have observed this progress in the results of many cases; but the reticulated appearance was remarkably distinct in the septum of a man who had died with symptoms of contagious fever, and in whom this effect had succeeded to the effusion of pellucid fluid. This had taken place both beneath the arachnoid membrane and in the ventricles; in consequence of the latter of which, the mesolobe was elevated from the fornix, and the cerebral matter of the septum was so stretched as to form the network appearance which I have noticed. In this instance the gentlest application of the finger of one of the spectators produced the breach of the reticula and the retraction of the fibres, so as to form the elliptical aperture, and complete the communication. I have since been informed by Mr. Staff-surgeon Schetky, that he has lately seen an equally well-marked instance of the reticular septum, in consequence of watery effusion, in the brain of a man who had died of very general organic disease.

The last morbid appearance which I shall notice, as presented by the arachnoid membrane, is another instance of an alteration taking place in it, but depending on a diseased action which originates in another part of the encephalic organ. Instead of the smooth and glistening aspect which characterizes the sound condition of this envelope, the pathographical observer remarks that it has become unusually dry, and seems to be no longer lubricated with the fluid which exudes from its non-adherent surface. I do not at present remember that this change in the arachnoid membrane has been mentioned by authors on the subject; but I am quite satisfied of this fact, that it has never been remarked to be connected with a particular condition of the central surface of the brain. I have invariably found it in the true hydrencephalus of children, and (so far as my observation bears me out,) it indicates the presence of water in the cerebral cavities. I do not at present remember a well-marked case of this disease, the acute hydrencephalus, in which I did not first recognize the dry, dull, and lustreless appearance of the arachnoid membrane, and find, at the same time, a quantity, more or less considerable, of limpid fluid in the ventricles. My limits will not permit me to offer any observations on the link which seems thus to connect the actions of two different parts of the same organ; but, according to the usual principles of medical reasoning, I believe it will generally be considered as an example of the transference or modification of a natural action, in consequence of the excessive degree of a diseased one. The symptoms attending this appearance are abundantly well known, and require, on that account, no notice from me; but it might, perhaps, lead my readers to misunderstand what I have advanced concerning this state of the arachnoid membrane, if I failed to notice certain discriminating circumstances with which it is accompanied.

First, then, it might be understood that, whenever the cerebral cavities are expected, from the symptoms during life, to contain fluid at death, then the arachnoid membrane must be dry, dull, and destitute of its ordinary glistening aspect. This however, it might be remarked, is contradicted, both by what is generally known on the subject, and likewise by what I have myself said in a previous part of this paper. Fluid may be found in the cavities formed by the central surface, and the arachnoid be unchanged in its appearance; or it may exhibit the appearance which I have described when speaking of the aqueous state of the pia mater. There is, therefore, a certain order of affections in which the arachnoid membrane retains its shining aspect, and there are others in which this is no longer observed. The latter is what takes place in the acute hydrencephalus, and is, in truth, the only pathographical phenomenon which particularly distinguishes this disease from the other morbid conditions in which fluid is found in the ventricles.

Secondly, a great number of affections of the head have a common termination in effusion on the two surfaces of the brain, the convoluted or exterior, and the central, or that of the cavities. We observe these in persons of all ages and habits,—in the young and in the old, in the robust in certain circumstances, and in the feeble and strumous habits; and we recognize them as a sequel to many different, or even opposite, maladies,—to contagious fever, to dropsy, to diseases of the organs of respiration and circulation, to traumatic fevers, &c. We observe this effusion taking place in periods of very short duration, and sometimes only in the course of many weeks or months. Lastly, we see it, in some instances, attended with very characteristic and formidable symptoms, and at other times producing so little effect on the other functions that the most sagacious observation cannot, in certain circumstances, be led to suspect it. Are these effusions the same with that of the acute hydrencephalus? If they are not, in what respect do they differ from it? I cannot indulge in the hope that I am to explain this difficulty entirely; but I trust that the following remarks will tend to elucidate it in some degree.

Blood may be extravasated on the convoluted surface of the brain, in several different circumstances. That which is the effect of external violence properly comes under the class of injuries of the head, and does not therefore form a necessary part of my duty to consider. When it takes place spontaneously, it produces that assemblage of effects on the functions of sensation and locomotion, to which physicians have given the name of apoplexy. It may occur in almost any point of this surface, but I think it is most frequently observed somewhere along the inferior region. The anfractuosities contiguous to the fissure of Sylvius are very generally occupied with bloody extravasations; and this may be attributed to the vicinity of a numerous cluster of vessels. The *white perforated spot* of Vicq d'Azyr, which is remarkable for admitting a great number of vessels from the sylvian artery into the substance of the brain, has been observed, by Joseph and Charles Wenzel, of Vienna, to be more frequently affected than any other region of the convoluted surface.

I have frequently seen the anfractuosities between the cerebellic folia considerably distended with effused blood, and an extraordinary number of blood-drops on the exterior surface of the annular protuberance.

Similar appearances may be observed on the central surface of the brain; and there is scarcely a single body found in that surface which has not been recorded by some pathologist as the seat of bloody effusion. The parts, however, most frequently affected are the striated body and optic thalamus. Whether these hemorrhagic events occur on the exterior or the interior surface of the brain,—whether they take place on the optic thalamus or the striated body, or any other part,—they are uniformly attended with one of two effects. If the quantity be considerable,—if it be effused suddenly,—if active remedies be not used,—or if, when used, they be inefficacious, then a mortal compression will speedily kill the patient, and the appearances on dissection will be nearly one or other of those which I have already noticed. If, however, by the proper use of remedies, the effused blood be prevented from acting so long and so completely as to extinguish life immediately, then further changes are found to take place; and these go on until they have so altered the natural structure, that life can no longer be continued. The effused blood breaks down, softens, and disorganizes the surface, which, instead of presenting its ordinary polished and rounded appearance, becomes rough, irregular, soft, and pulpy. This change, which may take place in the course of about from seventy hours to eight days, has been noticed by Mr. John Hunter and some other authors. It has generally been considered as a species of suppuration; and perhaps this action may succeed to some inflammatory state of the cerebral matter, induced by the presence of the blood, which then acts like a foreign body. I am, however, very doubtful if we can suppose the cerebral matter ever to undergo the suppurative process, unless in consequence of external injury; for I believe that spontaneous inflammation, taking place in this organ, proves fatal long before it passes into the suppurative stage. I am aware that, in advancing this opinion, I expose myself to the charge of contradicting the descriptions given by very high authorities; but the observations of Dr. Baillie, the writer to whom I allude, have, I conceive, been derived either from cases which were really and truly extravasations of blood, or the appearances produced by the sequelæ of injuries of the brain.

Effusion of blood into the cerebral substance occurs very frequently in the grey matter of the convolutions. This matter is arranged in fibrous lines, the direction of which is perpendicular to that of the convoluted surface, and the blood is generally effused in the interstices between these lines. The effusion, however, is seldom restricted to the grey matter, but in most cases penetrates into the white matter of the hemispheres. A very common situation for this event is the grey and white substance corresponding to the white perforated spot of Vicq d'Azyr. This part, which is situated at the interior extremity of the fissure of Sylvius, corresponds to the matter which is found to form the striated body; and, when examined more carefully, it is found that the vessels which enter at this part of the organ continue their course into that peculiar substance to which the name of the Capsule of Reil has been given. The whole striated body, the capsule of Reil, and the grey matter of the sylvian fissure, are on this account, and from the vicinity of these vessels to the trunk of the internal carotid, exceedingly vascular; and it cannot be matter of wonder that hemorrhagic effusion should be most frequently observed in this situation.

Next to the striated body, the annular protuberance is the most frequent seat of that morbid change which we are at present considering. The mode in which the extravasation takes place depends on the structure of this object. It consists of a twofold series of fibrous lines, the direction of which is perpendicular to each other. One set is arranged in the longitudinal, the other in the transverse, direction of the body. It is in the interstices of the latter order of lines that the bloody effusion is most frequently observed. A very well marked case of this kind occurred to my observation about two years ago; and in this blood was found so copiously and regularly deposited between the transverse fibres of the protuberance, that an eminent medical friend thought it worth recording by an oil painting. Regarding the effects of these changes of the protuberance on the system at large, Bichat says, in a manner equally brief and expressive, that although numerous cases of lesion of the brain proper and the cerebellum, without affecting the other organs, are on record, yet he knew of none of the protuberance which was not fatal.

In the substance of the brain, bodies of a greater consistence than the surrounding parts are sometimes met with in the course of dissection. To these the general name of tumors has been applied. The most rational view of their origin and nature would lead us to deem them, in general, to be cerebral matter considerably changed by some morbific cause, and no longer presenting its usual organized appearance; but generally much harder, resembling sometimes cheese, sometimes soft cartilage. The observations and dissections of the two Wenzels lead to the following results:—that the brain proper may be the seat of disorganized portions of three different kinds; 1st, a tumor of an adipose nature; 2d, a substance of an osseous nature; 3d, a substance resembling scirrhus. Among the great number of brains which these anatomists have examined, only two presented the adipose degeneration. These were found toward the exterior or convoluted surface, but penetrated pretty deep into the substance of the organ. The aspect of the interior structure is subcinereous, its consistence solid and firm, approaching in some parts to bone. Of the scirrhous degeneration, these anatomists met with only two examples, both of which were situated on parts connected with the central surface; an important mark of difference from that of the adipose tumor, so far as we hitherto know. The ossific change is infinitely less common. With regard to the exclusive seat of all these disorganized portions, it is a general fact that none have been hitherto recognized in the cerebellum.

There is, perhaps, nothing in the whole of pathological science so puzzling and contradictory as the semeiography of these bodies. I have had occasion to know or observe several of them which seemed to have originated under a great variety of different circumstances. The writings of pathologists are full of cases in which tumors had been found in the brain after death, when no morbid appearance (at least not of that kind, nor in that part,) had been anticipated;—cases in which, after obstinate and violent cephalæa, substances had been found in the brain, which had first irritated and then compressed it;—and cases in which substances very unlike each other had produced very different effects on the other functions. I have in my collection two small bodies of an irregular form, but not exceeding the size of a small bean, which were found in the anterior lobes of the brain of a young person who had died of chorea, a disease very rarely fatal. It is well known that most of these tumors, as they have been named, give rise first to epileptic, and then to apoplectic symptoms; but it is remarkable that, in very many cases of epilepsy, no such appearance has been found. I knew very intimately the subject of one of these examples of disorganized brain denominated tumor, in whom the first symptoms appeared in the course of a very severe febrile disease, accompanied with affection of the head,—acute pain, double vision, vertigo, and deafness alternating with *tinnitus aurium*. Yet life was continued in exceeding good health for more than a year; and death took place in consequence of phthisis, fully eighteen months after the original malady.—*(Edinburgh Med. and Surg. Journal.)*

